# A Case of Nocardia cyriacigeorgica Infection and Literature Review

**DOI:** 10.7759/cureus.87189

**Published:** 2025-07-02

**Authors:** Jing Li, Yong Zhou, Nannan Zou, Min Chen

**Affiliations:** 1 Clinical Laboratory/Molecular Biology, Qingdao Sixth People's Hospital, Qingdao, CHN; 2 Infectious Diseases/Clinical Medicine, Qingdao Public Health Clinical Center, Qingdao, CHN; 3 Clinical Laboratory/Clinical Microbiology, Qingdao Public Health Clinical Center, Qingdao, CHN; 4 Clinical Laboratory/Morphology, Qingdao Public Health Clinical Center, Qingdao, CHN

**Keywords:** cough, hiv, mycobacterium, nocardia cyriacigeorgica, nocardia species

## Abstract

*Nocardia* bacteria primarily enter the human body through the respiratory tract or open wounds, leading to suppurative infections. These infections are more prevalent in individuals with compromised immune systems and can affect the lungs, resulting in pulmonary nocardiosis. The bacteria may also disseminate via the bloodstream to adjacent tissues or infect various organs. Clinical manifestations, physical signs, and imaging findings of nocardial pneumonia lack specificity. Additionally, *Nocardia* grows slowly and is often overgrown by faster-growing bacteria in sputum cultures, making it difficult to isolate. As a result, clinical misdiagnosis and missed diagnosis are common. With the growing number of immunocompromised individuals, the incidence of nocardial infections has been increasing. Improving laboratory personnel's awareness of this pathogen and enhancing their technical capabilities are crucial for accurate and timely clinical diagnosis.

A 34-year-old female patient was reported to have contracted pneumonia caused by *Nocardia cyriacigeorgica*. The patient experienced a cough and sputum production without a clear cause 20 days prior. Despite clinical empirical treatment, the cough and sputum persisted, and there was also a high fever accompanied by chills and shivering. Metagenomic next-generation sequencing (mNGS) results of the bronchoalveolar lavage fluid showed that it was caused by *Nocardia cyriacigeorgica*. After four days of sputum culture, bacterial colonies were observed and subsequently identified by matrix-assisted laser desorption ionization time-of-flight mass spectrometry (MALDI-TOF-MS) as *Nocardia cyriacigeorgica*. This patient received a combined treatment of compound sulfamethoxazole/trimethoprim and linezolid. Soon, his condition improved, and he was discharged from the hospital. During the two-month follow-up examination, it was observed that the lesion had been largely absorbed and the affected area had significantly reduced in size. The patient no longer experienced coughing or phlegm production.

Following a comprehensive review of the clinical data of this case and relevant literature, we aim to improve the capacity of laboratory personnel in cultivating and identifying this rare bacterial pathogen. Furthermore, this study seeks to emphasize to clinical practitioners that bronchoalveolar lavage fluid should be collected for mNGS when pulmonary *Nocardia* infection is suspected, which can enhance diagnostic accuracy, facilitate early detection and timely intervention, and ultimately alleviate the burden on patients.

## Introduction

*Nocardia cyriacigeorgica* belongs to the *Nocardia* genus. It was first reported by Yassin in 2001 [1]. In 2003, this strain was isolated from the bronchial secretions of a patient with chronic bronchitis [1]. Since then, this bacterium has been detected in many countries and regions around the world. In recent years, with the increase in patients with immune dysfunction, blood system diseases, organ transplant patients using immunosuppressants, and long-term use of hormones, the incidence of *Nocardia* infection has increased [2]. A total of 70%-80% of *Nocardia* infections present with early symptoms of pulmonary infection. The clinical manifestations of pulmonary *Nocardia* infection are not specific and are prone to misdiagnosis and missed diagnosis. Therefore, the ability of clinical laboratories for culture and identification is very important [3]. If not treated promptly, the mortality rate is high. This article analyzes a case of pulmonary *Nocardia* infection caused by *Nocardia cyriacigeorgica* treated at the Public Health Clinical Center of Qingdao, summarizes the experience in identifying and drug sensitivity testing of *Nocardia cyriacigeorgica*, and assists in the clinical diagnosis of *Nocardia cyriacigeorgica* disease.

## Case presentation

Patient information

The patient is a 34-year-old female who experienced an onset of coughing 20 days ago without any apparent cause. The cough was severe, accompanied by right-sided chest pain, expectoration of yellow, sticky sputum in large amounts, without fever, chills, or fear of cold at the beginning. She did not experience nausea, vomiting, abdominal pain, or diarrhea. Initially hospitalized at a local facility, she was administered anti-inflammatory drugs (details unspecified) for pneumonia treatment. After one week, her symptoms showed improvement, but recently she has started coughing again, accompanied by a small amount of sputum. A fever began one day ago, peaking at 39.1℃, accompanied by chills. Human immunodeficiency virus (HIV) antibody tests at the local hospital were positive. Seeking further diagnosis and treatment, she arrived at our hospital. The emergency department admitted her to the infectious diseases department with a diagnosis of acquired immunodeficiency syndrome (AIDS). Examination revealed thickened respiratory sounds in both lungs, with audible wet rales. Her heart rate was 112 beats per minute. Her hemoglobin level was 81 g/L. The preliminary diagnosis indicated (1) AIDS, (2) severe pneumonia, and (3) moderate anemia.

The process of examination, inspection, diagnosis, and treatment

The total number of white blood cells significantly increased (11.36 x 10^9^/L), accompanied by an increase in absolute neutrophil count (10.51 x 10^9^/L), indicating the highest possibility of acute bacterial infection. CRP (228.10 mg/L) and procalcitonin (PCT) (25.09 ng/ml) were extremely elevated, consistent with the characteristics of sepsis or severe systemic infection. Based on this situation, the patient received combined treatment with meropenem and azithromycin. The patient's T-cell tuberculosis infection test was weakly positive (+), indicating the possibility of tuberculosis infection. Enlarged mediastinal lymph nodes and bilateral lung infection lesions (in the lower right lobe) suggest the possibility of tuberculosis dissemination. However, the culture for *Mycobacterium tuberculosis* was negative, and the result for *Mycobacterium tuberculosis* deoxyribonucleic acid (DNA) was also negative, basically ruling out the possibility of pulmonary tuberculosis. The Epstein-Barr virus (EBV) DNA load was 4.78×10³ copies/ml, and the cytomegalovirus (CMV) DNA was 3.24×10⁵ copies/ml, indicating the coexistence of those two virus infections. Patients infected with the EBV are less likely to have symptoms such as coughing and expectoration. For patients infected with CMV, the cough is usually dry. Therefore, in clinical practice, to identify the pathogen causing pneumonia, lung lavage fluid from the patient was collected for metagenomic next-generation sequencing (mNGS) testing, and sputum specimens were collected and sent to the microbiology laboratory for smear examination and culture to search for the pathogen. The detection of *Aspergillus niger* galactomannan antigen and (1,3)-β-*Aspergillus niger* galactomannan antigen were both negative, indicating no fungal infection. Severe hypoproteinemia (albumin: 21.5 g/L, prealbumin: 19.6 mg/L) and albumin/globulin (A/G) ratio reversed (0.8), accompanied by low calcium (1.81 mmol/L), required nutritional support treatment. The patient presented with anemia, hypoproteinemia, and a poor diet, for which amino acids and Shengxuebao mixture were prescribed orally. The patient had a history of HIV infection (HIV ribonucleic acid: 3.46×10⁴ copies/ml). The patient's T helper cell (TH) lymphocytes (CD4) were 2 cells per microliter, showing a significant decrease. The T suppressor cell (TS) lymphocytes (CD8) were 160 cells per microliter, with a TH/TS ratio of 0.01, indicating a significant inversion. This suggests that the patient's immune system was imbalanced and her immune function was disordered. The patient was advised to start taking antiretroviral drugs as soon as possible because she had not used antiviral drugs in a standardized manner and had taken many medications on her own, such as tenofovir disoproxil fumarate tablets, lamivudine tablets, and efavirenz tablets, and had stopped taking the medication five years ago. Based on this, we assessed that the patient had a relatively high risk of developing resistance to these antiviral drugs. Therefore, we recommend that this patient receive treatment with other antiretroviral drugs (elvitegravir tablets). The laboratory test results are shown in Table 1.

**Table 1:** Laboratory test results. CMV: cytomegalovirus; EBV: Epstein-Barr virus; FDP: fibrinogen degradation product; TH: T helper cells; TS: T suppressor cells.

Tests (reference range)	Results
Hemoglobin (120-160 g/L)	81 g/L
White blood cells (4-10x10^9^/L)	11.36x10^9^/L
Neutrophils (2-7.7x10^9^/L)	10.51x10^9^/L
C-reactive protein (0-10 mg/ml)	228.10 mg/ml
Erythrocyte sedimentation rate (0-15 mm/h)	119 mm/h
HIV-1 RNA (<3.0E1 copies/ml)	3.46E4 copies/ml
Procalcitonin (≤0.05 ng/L)	25.09 ng/L
Free triiodothyronine (1.8-4.2 pg/ml)	1.00 pg/ml
Ferritin (27-375 ng/ml)	1869.39 ng/ml
Prothrombin time (10-14 seconds)	15.40 seconds
Fibrinogen (2-4 g/L)	5.15 g/L
D-dimer (0-0.5 mg/L)	1.25 mg/L
FDP (0-5 ug/mL)	5.30 ug/mL
Basophil (0-0.1x10^9^/L)	0.08x10^9^/L
Platelet count (100-300x10^9^/L)	228x10^9^/L
Direct bilirubin (0-6.8 umol/L)	10.6 umol/L
Aspartate aminotransferase (15-40 U/L)	41 U/L
Alkaline phosphatase (53-128 U/L)	199 U/L
Glutamyl transpeptidase (0-38 U/L)	86 U/L
Total protein (66-88 g/L)	49.7 g/L
Albumin (35-52 g/L)	21.5 g/L
Prealbumin (220-400 mg/L)	19.6 mg/L
High density lipoprotein (0.8-1.8 mmol/L)	0.18 mmol/L
Cholinesterase (5100-1170 U/L)	890 U/L
Glomerular filtration rate (80-120)	129.83
Magnesium (0.75-1.05 mmol/L)	1.04 mmol/L
Calcium (2.1-2.9 mmol/L)	1.81 mmol/L
Retinol-binding protein (20-70 mg/L)	5.50 mg/L
Immunoglobulin A (0.7-4 g/L)	5.27 g/L
CMV DNA (<4.00E2 copies/ml)	3.24E5 copies/ml
EBV DNA (<4.00E2 copies/ml)	4.78E3 copies/ml
TH lymphocytes (CD4) (410-1590 cells/ul)	2 cells/ul
TS lymphocytes (CD8) (190-1140 cells/ul)	160 cells/ul
TH/TS ratio (0.7-2.8)	0.01

Pathogen detection results and drug sensitivity

**Figure 1:**
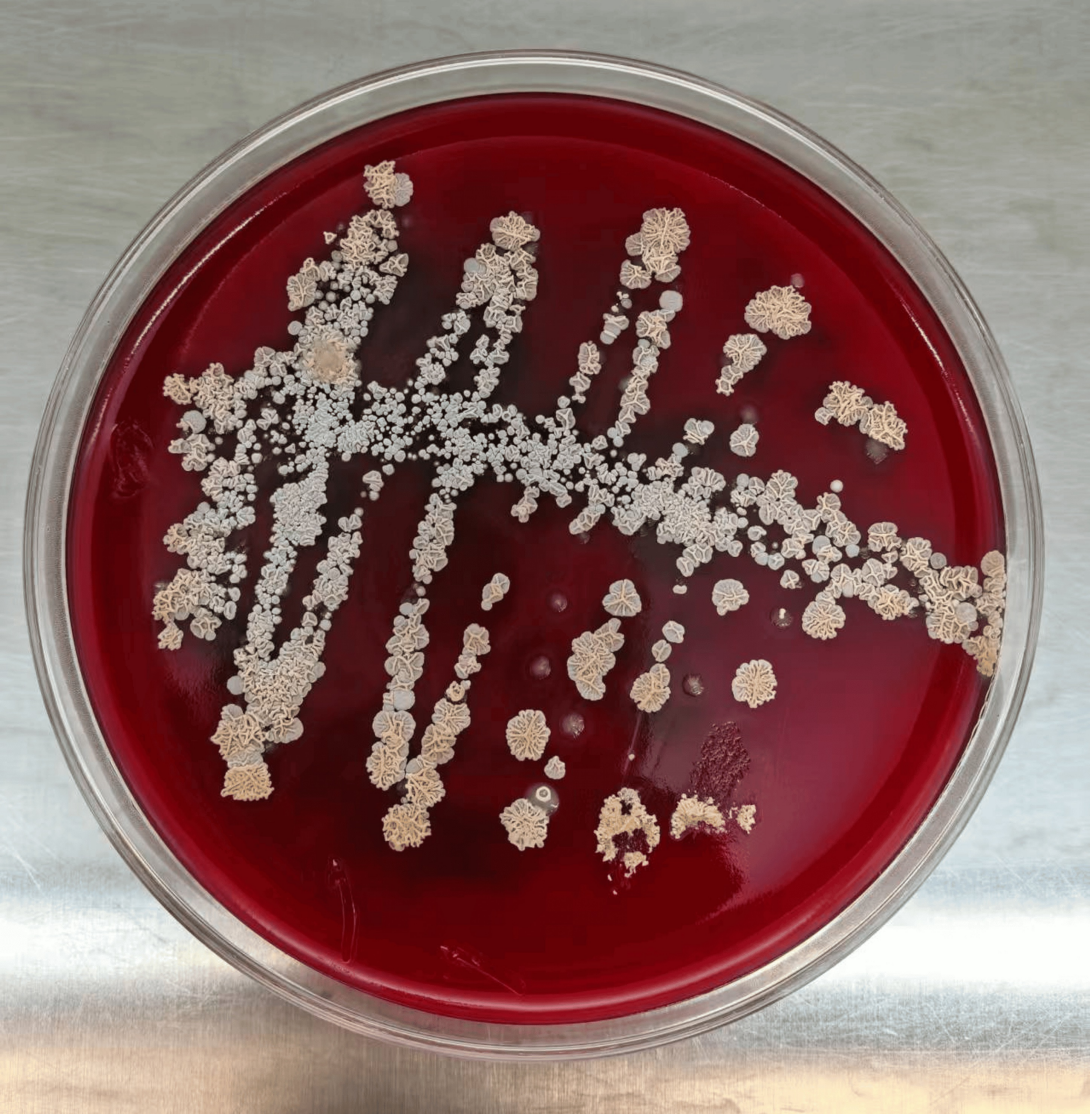
Positive picture of sputum culture. On a medium containing 5% sheep blood agar, after four days of incubation at 37℃, powdery white, dry, wrinkled, and slightly raised colonies appeared.

**Figure 2:**
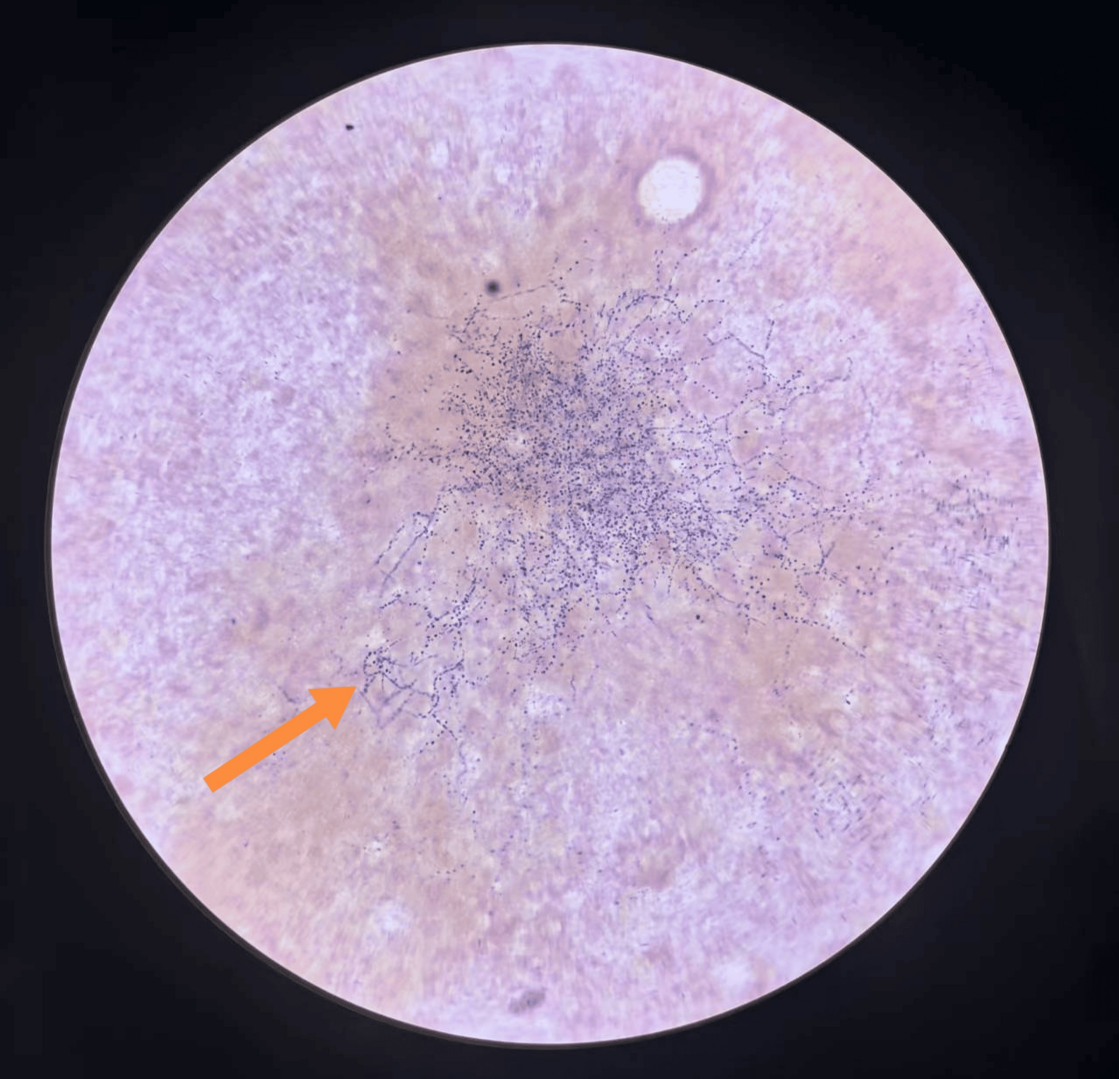
Gram-positive image. The cultivated strain was stained with Gram's method. Under the oil microscope (1000x), Gram-positive branched bacilli and bead-like structures were observed.

**Figure 3:**
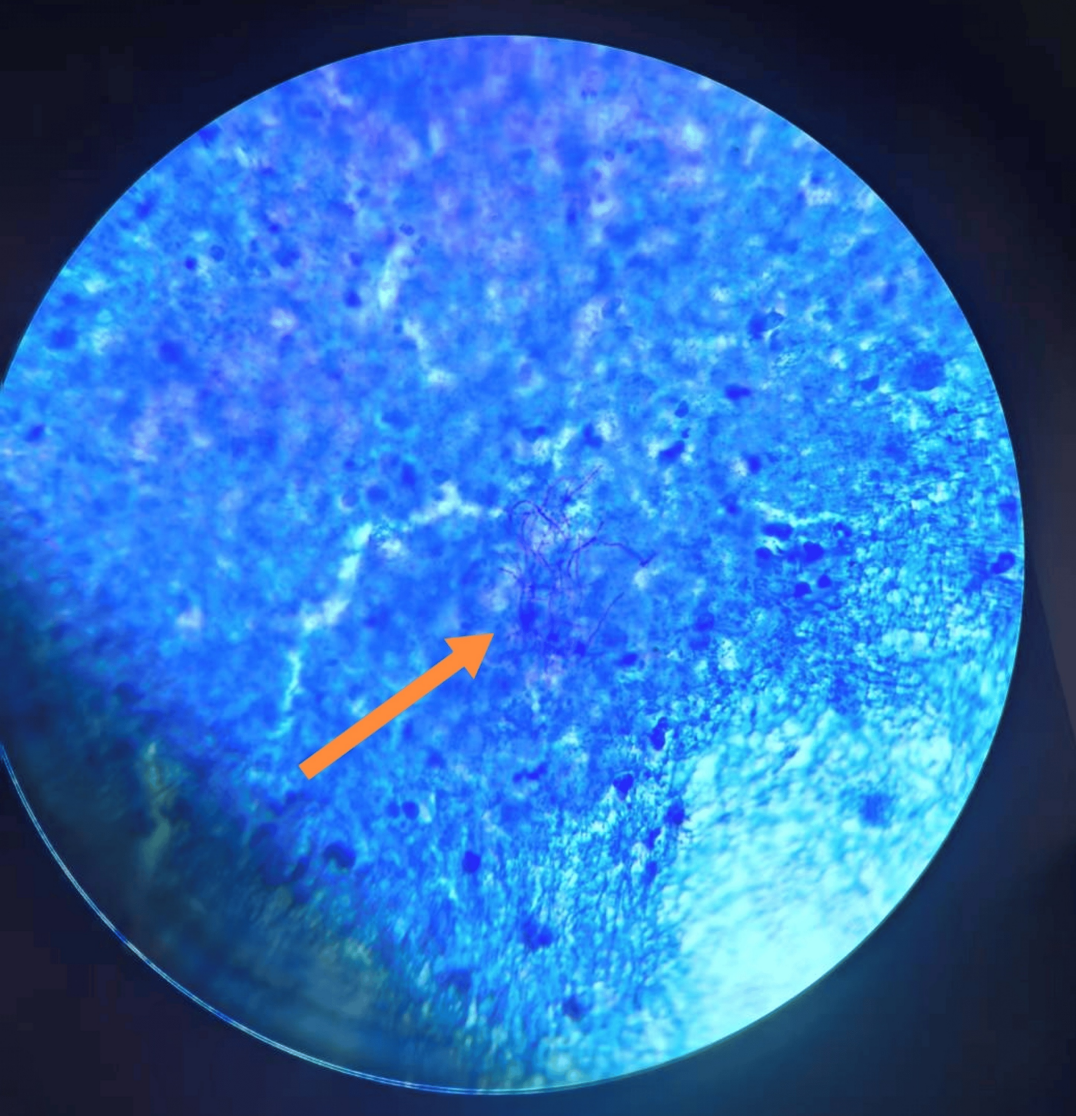
Weak acid-fast staining-positive picture. Using acid-fast staining (with a 1% dilute sulfuric acid solution for decolorization), under the oil microscope (1000x), weak acid-fast staining-positive bacilli were observed.

**Figure 4:**
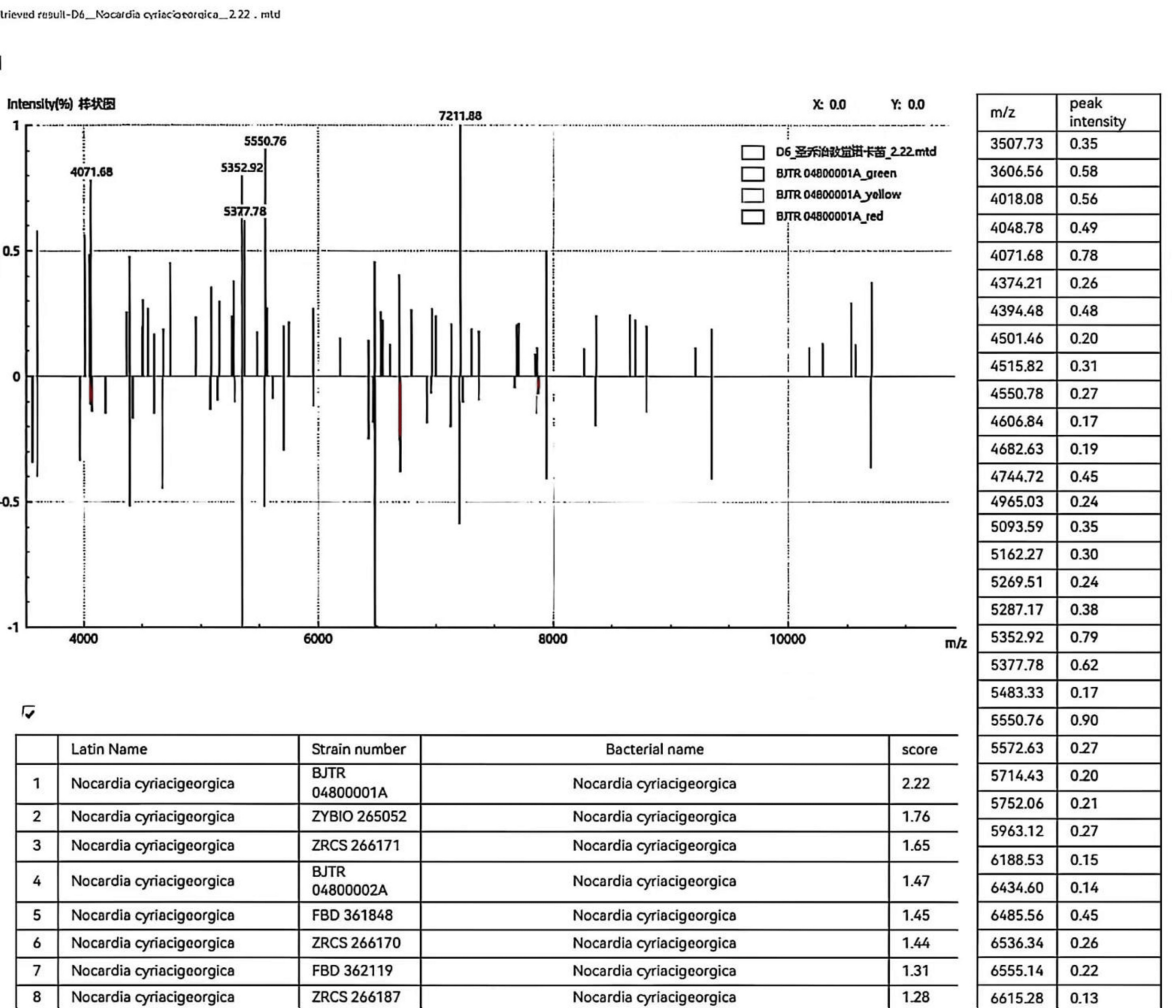
The patient's MALDI-TOF-MS results. Through peak intensity analysis, it was determined that the pathogen was *Nocardia cyriacigeorgica*. MALDI-TOF-MS: matrix-assisted laser desorption ionization time-of-flight mass spectrometry.

After four days of sputum culture (37°C), powdery white, dry, wrinkled, and slightly raised colonies appeared on the blood agar plate (containing 5% sheep blood) (Figure 1). We prepared the cultivated colonies into smears, performed Gram staining on the smears, and observed them under a 1000x microscope. We found Gram-positive bacilli, with the hyphae branching at a 90-degree angle and some of the hyphae presenting a string-like appearance (Figure 2). We performed weak acid-fast staining on the smear (with 1% sulfuric acid solution as the decolorizer), and under a 1000x microscope, we observed weakly acid-fast-positive bacilli (Figure 3). The strains we cultivated were identified as the cyriacigeorgica type of *Nocardia* by Zybio EXS1000 (Zhongyuan Huiji Biotechnology Co., Ltd., Chongqing, China) MALDI-TOF-MS (matrix-assisted laser desorption ionization time-of-flight mass spectrometry) (Figure 4). The drug sensitivity results are shown in Table 2, with sensitivity to trimethoprim sulfamethoxazole, linezolid, imipenem, tobramycin, amoxicillin clavulanic acid, and amikacin. The mNGS results of bronchoalveolar lavage fluid showed the presence of *Nocardia cyriacigeorgica*, human gammaherpesvirus, human betaherpesvirus, human alphaherpesvirus, and *Streptococcus pneumoniae* infections (Table 3), and the results showed resistance to macrolide drugs. The patient received meropenem and azithromycin for three days. After the mNGS results were available, it was found that the patient was infected with *Nocardia* and had resistance to macrolide antibiotics. The patient was administered a combination therapy of sulfamethoxazole/trimethoprim and linezolid. Sulfamethoxazole/trimethoprim was started at an initial dosage of 15 mg per kilogram of body weight per day, delivered intravenously in three divided doses. Subsequently, the dosage was adjusted to 10 mg per kilogram of body weight per day, administered orally for a total duration of six months. The patient also received linezolid at a dosage of 600 mg per administration, given orally every 12 hours, for a total duration of six months. The results of the follow-up examination two weeks later were as follows: white blood cell count of 4.78 × 10^9^/L, hemoglobin at 90.00 g/L, neutrophil count of 2.06 × 10^9^/L, and procalcitonin at 0.43 ng/ml. The patient's infection status had improved, and there were no longer symptoms of coughing, sputum production, or fever. After discharge, the patient continued to take sulfamethoxazole/trimethoprim and linezolid orally for six months. During the two-month follow-up visit, the patient's lesion shrank and symptoms disappeared.

**Table 2:** The results of Nocardia cyriacigeorgica antibiotic sensitivity test. The drug sensitivity test results showed that this patient was sensitive to trimethoprim-sulfamethoxazole, linezolid, imipenem, tobramycin, amoxicillin-clavulanate, and amikacin. MIC: minimum inhibitory concentration.

Antimicrobial	MIC (mg/L)	Sensitivity
Trimethoprim sulfamethoxazole	≤2/38	S
Linezolid	≤8	S
Imipenem	≤4	S
Tobramycin	≤4	S
Amoxicillin clavulanic acid	≤8/4	S
Amikacin	≤8	S

**Table 3:** The patient's mNGS results. The metagenomic next-generation sequencing (mNGS) results of bronchoalveolar lavage fluid showed the presence of Nocardia cyriacigeorgica, human gammaherpesvirus, human betaherpesvirus, human alphaherpesvirus, and Streptococcus pneumoniae infections. The sequence number of Nocardia cyriacigeorgica was the highest, at 6640, indicating that this bacterium was the main pathogen causing infection in this patient.

Name of the pathogen	Sequence number	Degree of certainty in identification
Nocardia cyriacigeorgica	6640	99%
Human gammaherpesvirus	4846	99%
RV-B14	1645	99%
Human betaherpesvirus	925	99%
Rhinovirus B	335	99%
Human alphaherpesvirus	43	99%
Streptococcus pneumoniae	15	99%
Mycobacterium tuberculosis complex	Not detected	Not detected
Non-tuberculous mycobacteria	Not detected	Not detected
Fungi	Not detected	Not detected
Parasites	Not detected	Not detected

## Discussion

*Nocardia* is a Gram-positive aerobic bacterium. It is a non-human colonized opportunistic pathogen, mostly saprophytic bacteria, and widely exists in soil, air, grass, and decayed plants [4]. So far, 791 strains of *Nocardia* have been identified, of which 119 species of *Nocardia* have been confirmed in the literature, and 54 species of *Nocardia* are related to human infection. *Nocardia* does not belong to the normal flora of the human body, so it will not show endogenous infection. As long as this bacterium is cultivated in the laboratory, it is generally pathogenic [5,6]. In recent years, with the increase in patients with immune function defects, blood system diseases, immunosuppressive agents after organ transplantation, and long-term hormone use, the incidence of nocardiosis has increased. A total of 70%-80% of the early symptoms of nocardiosis are pulmonary infection [2]. Pulmonary nocardiosis has no specific clinical manifestations, and its imaging manifestations are diverse. Nodules, patches, and consolidation shadows are common, and even cavities, masses, pleural lesions, etc., are noted. It is difficult to make an early diagnosis, and it is often misdiagnosed as pneumonia, pulmonary tuberculosis, invasive fungal disease, and lung cancer. The level of laboratory culture and identification is very important. If it cannot be diagnosed and treated on time, the prognosis is poor, and the mortality rate is high (about 41%). *Nocardia* pneumoniae can also spread through blood and lead to systemic organ infection, even combined with brain abscess, skin and soft tissue infection, etc. [7,8]. Etiological examination is the gold standard for the diagnosis of *Nocardia* infection. It grows slowly. Under the microscope, it is slender, rod-shaped, branched, usually beaded, with irregular colony morphology, wrinkles, and weak acid resistance positive. It takes four to six weeks to obtain satisfactory results when cultured in ordinary medium at 37℃ [9-11]. Moreover, up to now, less than 1% of bacteria can be identified by isolation and culture. Microscopic observation can be used for morphological identification, but its sensitivity and specificity are low. Polymerase chain reaction (PCR) cannot detect unknown and highly variable pathogens [12-15]. At present, the most commonly used methods for identifying *Nocardia* are MALDI-TOF-MS and mNGS. MALDI-TOF-MS technology is based on the difference in mass charge ratio of different microbial proteins. The protein fingerprints of a single strain are compared with those in the database, and the identification results are obtained [16]. The technology of mNGS has a satisfactory diagnostic value compared to conventional methods in nocardiosis diagnosis and could assist clinical decision-making with minimized turnaround time. It can also detect mixed infections and drug resistance, assisting clinicians in making early diagnoses and providing timely treatment for patients. The sputum culture (37°C for four days) results of this patient showed white, dry, wrinkled colonies on a blood agar plate (containing 5% sheep blood). Microscopically, Gram-positive rods were observed, presenting as branching, beaded patterns. Upon additional weak acid-fast staining, weakly acid-fast, branching, multi-directional filaments were visible under the microscope. It was identified as *Nocardia cyriacigeorgica* by MALDI-TOF-MS. The results of mNGS of this patient's bronchoalveolar lavage fluid indicated that *Nocardia cyriacigeorgica* was consistent with the MALDI-TOF-MS identification, and the mNGS results suggested mixed infection in the patient and resistance to macrolide antibiotics.

*Nocardia* infection is not common in clinics, and there is a lack of multi-center drug sensitivity test data at home and abroad. In recent years, reports show that the resistance rate of *Nocardia* to sulfonamide antibiotics in vitro is on the rise [17,18]. But when the dose is enough, it can still be effective in vivo. Linezolid has an obvious therapeutic effect on all *Nocardia* strains. According to the medication recommendations of ABX guidelines and febrile disease, the preferred treatment is still sulfamethoxazole/trimethoprim, which can be used in combination with other drugs [19]. Our laboratory used the minimum inhibitory concentration (MIC) method to provide some in vitro drug sensitivity test data for clinical use. The results showed that sulfamethoxazole/trimethoprim, linezolid, imipenem, tobramycin, amoxicillin-clavulanate, and amikacin were all sensitive. Therefore, the patient was administered a combination therapy of sulfamethoxazole/trimethoprim and linezolid. The initial dosage of sulfamethoxazole/trimethoprim was 15 mg per kilogram of body weight per day, delivered intravenously in three divided doses. Subsequently, the dosage was adjusted to 10 mg per kilogram of body weight per day, administered orally for a total duration of six months. The patient also received linezolid at a dosage of 600 mg per administration, given orally every 12 hours, for a total duration of six months. The patient was discharged from the hospital two weeks later. During the patient's follow-up examination two months later, the clinical symptoms disappeared, and the imaging results showed that some of the lung lesions had been absorbed. The treatment was found to be quite effective.

## Conclusions

In summary, the clinical manifestations of pulmonary nocardiosis lack specificity and are challenging to differentiate from other pulmonary pathologies. The conventional laboratory culture method, hindered by the slow growth rate of *Nocardia* species, rarely yields definitive results within a 48-hour timeframe. Furthermore, this method is susceptible to interference from other bacterial colonies on blood culture media, often resulting in false-negative outcomes. With technological advancements, mNGS demonstrates superior sensitivity and reduced detection time compared to traditional laboratory techniques. Additionally, mNGS outperforms conventional microbial culture in identifying mixed infections and can provide comprehensive antimicrobial resistance profiles. These advantages are particularly significant for the early diagnosis and therapeutic intervention of nocardiosis. Consequently, we recommend implementing a multimodal diagnostic approach, with particular emphasis on mNGS technology, to facilitate rapid pathogen identification in patients with suspected nocardiosis infection.
